# The Bibliometric Evolution of Neurosurgery Publications From 1977 to 2023

**DOI:** 10.1227/neuprac.0000000000000128

**Published:** 2025-01-30

**Authors:** Michael M. Covell, Seyed Farzad Maroufi, David Kurland, Karl L. Sangwon, Bethsabe Romero, Marc Moisi, Johnny Delashaw, Meic Schmidt, Christian A. Bowers

**Affiliations:** * School of Medicine, Georgetown University, Washington, District of Columbia, USA;; ‡ Bowers Neurosurgical Frailty and Outcomes Data Science Lab, Flint, Michigan, USA;; § Department of Neurosurgery, New York University, New York, New York, USA;; ‖ Eastern Virginia Medical School, Norfolk, Virginia, USA;; ¶ Hurley Neurological Center at Hurley Medical Center, Flint, Michigan, USA;; # Maine Medical Center, Portland, Maine, USA

**Keywords:** Authorship representation, Bibliometrics, Database utilization, Journal evolution, Neurosurgery

## Abstract

**BACKGROUND AND OBJECTIVES:**

*Neurosurgery* is a world leader in disseminating neurosurgical science. Although the literature has seen significant increases in academic collaboration, data science, and authorship diversity, the bibliometric evolution of *Neurosurgery* remains unknown. This study sought to elucidate bibliometric trends in *Neurosurgery* from 1977 to 2023.

**METHODS:**

Wolters Kluwer and Web of Science were used to identify all publications in *Neurosurgery* from 1977 to 2023. Database utilization was analyzed from 2007 to 2023 using abstract keywords. The BERTopic tool analyzed prevailing subspecialty themes. Publication types including clinical/case report/review were analyzed. Statistical analysis included exponential and linear regression.

**RESULTS:**

From 1977 to 2023, 29 755 publications containing 127 171 authors were published in *Neurosurgery*. From 1977 to 2023, the mean authorship count per publication exponentially increased (2.4 to 8.4, 2.0% annually) (*R*^2^ = 0.76), small authorship groups (≤ 5 authors) linearly decreased (90% to 37%) (*R* = 0.93), multinational authorship groups linearly increased (0% to 21.2%) (*R* = 0.95), and authorship affiliations per publication exponentially increased (1.3 to 4.6, 3.2% annually) (*R*^2^ = 0.65). From 1990 to 2023, female first authorship linearly increased (7.5% to 15.7%) (*R* = 0.93), Bachelor degree first authorship increased (0.6% to 12.5%), and MD/PhD senior authorship increased (6.2% to 24.7%), with more review articles and less case reports. Cerebrovascular topics dominated from 1980 to 2020, with spine topics most represented in the 2020s. Database utilization demonstrated an exponential, 4.6-fold increase from 2007 (0.38%) to 2023 (2.14%) (*R* = 0.63).

**CONCLUSION:**

*Neurosurgery* publications have demonstrated increasing authorship counts, authorship diversity, collaboration, and database utilization since the journal's 1977 inception, prominently publishing on topics in cerebrovascular and spine research.

**ABBREVIATION:**
BERT: Bidirectional Encoder Representations from Transformers.

The Congress of Neurological Surgeons published its first issue of *Neurosurgery* in 1977. Driven to benefit organized neurosurgery and members of the Congress, *Neurosurgery* has cemented its reputation as a leading medium to disseminate neurosurgical knowledge.^1^ Despite a consistent mission since its conception, the corpus of *Neurosurgery* publications has continually evolved to reflect modern biomedical research and offer novel science to its readership.

Bibliometric analyses offer novel insight into publishing trends, including the emergence of topics, authors, and study types specific to a particular field. Numerous bibliometric studies in neurosurgery have examined publishing behavior across the neurosurgical subspecialties, including spine, pediatrics, skull base, and neuro-oncology.^2-5^ Bibliometric analyses of neurosurgery journals have been performed to assess authorship contributions, citation count, and publication speed, but have been limited to single decades or narrow topics.^6-8^ Bibliometric analyses of neurosurgery journals have demonstrated increasing authorship counts and data utilization consistent with the emergence of “big data.”^9,10^ These trends may be concerning to some authors because database studies lack specific individual patient characteristics, do not account for variability in surgeon expertise, and do not include intricacies of neurosurgical care, such as operative time or medication doses.^9^ Increasing authorship counts has stimulated dialogue related to the integrity of authorship contributions and the seemingly increasingly low threshold to qualify for authorship.^10,11^

To date, no studies have specifically performed a bibliometric analysis to investigate publication trends in *Neurosurgery*. Examining the journal's bibliometric genotype may elucidate the neurosurgeon's role in emerging academic publishing trends in a leading neurosurgery journal. Understanding changes in authorship count, authorship demographics, and database utilization may glean insight into the direction of *Neurosurgery*'s publication corpus. This study sought to characterize the evolution of *Neurosurgery* publication content from 1977 to 2023.

## METHODS

### Data Sources

A complete list of publications in *Neurosurgery* from 1990 to 2023 was acquired from Wolters Kluwer (Wolters Kluwer N.V., Alphen aan den Rijn, Netherlands), the journal's publishing group, with permission from the Editor-in-Chief. The provided data set included publication title, edition, study type, authors (with affiliations/degrees), and publication year.

Scopus is an abstract and citation database of peer-reviewed literature including scientific journals and biomedical research (https://www.scopus.com/home.uri). Scopus was searched using the “Neurosurgery” source title to identify all records published in *Neurosurgery* and indexed in Scopus. A separate Scopus analysis was conducted to identify studies in *Neurosurgery* from 1977 to 1989. The Scopus and Wolters Kluwer data sets were compiled to capture the entire corpus of *Neurosurgery* from inception (1977) to 2023. Bibliometric parameters (title, year/edition, authors, author affiliations) were recorded, and publication abstracts were extracted from Scopus. Institutional Review Board approval was neither sought nor required as all data analyzed are publicly available. Patient consent and ethics committee approval were neither sought nor required because no protected health information or patient data were analyzed.

### Bibliometric Outcomes of Interest

From 1977 to 2023 in *Neurosurgery*, authorship counts per publication were investigated. Small authorship groups were defined as ≤ 5 authors. Authorship degree representation was investigated using conditional statements. Trends in authorship degree representation were analyzed using the Wolters Kluwer data set from 1990 to 2023. Labeled degrees included MD, DO, MBBS, MD/PhD, MD/MBA, PhD, or Bachelor Degree (BS, BA, BSc, BBA, BASc). Bachelor degree status served as a surrogate for medical student authorship. Female first authorship representation was investigated from 1990 to 2023. The Python package *gender_guesser*, previously deployed by other neurosurgery bibliometric analyses, was used to automate first authorship name recognition.^3,4,12^ *Gender_guesser* identifies names from a dictionary containing over 45 000 first names from the United States and India, as well as European and Asian countries, which are validated by native speakers of multiple countries.^13^ All first author names were labeled as female, mostly female (both counted as female), male, mostly male (both counted as male), or unknown. Unknown labeled names were not included in the analysis to avoid mislabeling. 1990 to 2023 was selected for the gender analysis to coincide with the degree representation analysis and because many publications in *Neurosurgery* before 1990 used author initials rather than full names. Previous bibliometric analyses of skull base and spine publications revealed minimal female authorship representation before 1990, supporting this methodological decision.^2-4^ Authorship affiliations were extracted from each publication. Country names were extracted from authorship affiliations. Multinational authorship was defined as authorship contributions from more than one country. International authorship was defined as publications without a single author from the United States. Analyses for authorship affiliations, multinational authorship, and international authorship were performed for publications from 1977 to 2023.

### Identifying Trends in Journal Content, Publication Type, and Database Utilization

Python Selenium library was used to automate the retrieval of PDF files from *Neurosurgery* beginning in 1977 and Meta Nougat (Meta Platforms, Inc.) to extract texts from all published articles. Articles were categorized by decade, and lexical analysis was performed to track keyword frequency and thematic evolution. The BERTopic tool, using Bidirectional Encoder Representations from Transformers (BERT) embeddings and clustering algorithms, was used to identify and analyze prevailing subspecialty themes across the collected documents. The Wolters Kluwer data set was used to analyze trends in publication type from 1990 to 2023. Each publication type was previously labeled by Wolters Kluwer, and the authors manually consolidated labels into abstract, anatomical, annual meeting related, award, book chapter, case report, clinical, commentary, correspondence, editorial, guidelines, legacy, review, and other. The Scopus data set was used to investigate database utilization in *Neurosurgery* from 2007 to 2023. This period was selected in conjunction with the emergence and growing popularity of database studies across the surgical literature. A comprehensive list of databases was compiled using the existing neurosurgical literature (Table 1).

**TABLE 1.** Outcomes Databases Included in Web of Science Query of *Neurosurgery* Publications From 2007 to 2023

• NSQIP—National Surgical Quality Improvement Program • TQIP—Trauma Quality Improvement Program • NIS—National Inpatient Sample • Nationwide Inpatient Sample (now NIS) • QOD—Quality Outcomes Database • N2QOD (now QOD) • NeuroPoint Alliance (now QOD) • National Readmissions Database • TriNetX • NCDB—National Cancer Database • NTDB—National Trauma Data Bank • KIDS—Kids Inpatient Database • SID—State Inpatient Database • SEER—Surveillance, Epidemiology, and End Results • PHIS—Pediatric Health Information System • PearlDiver • MarketScan • Premier Perspective

### Data Analysis

The R package *Bibliometrix* (https://www.bibliometrix.org/home/) was used to extract general journal information over the study period including publication output, author nationality, author affiliations, and keyword usage, as demonstrated in previous neurosurgical bibliometric analyses.^4,6,14,15^ Conditional statements on Microsoft Excel (Microsoft Corporation, Inc.) were developed to identify the degree of all authors published in *Neurosurgery* from 1990 to 2023. Figure creation and trend line analyses were conducted using Microsoft Excel, including linear and exponential regression. The data set may be provided by the authors on reasonable request.

## RESULTS

### Bibliometric Overview of Neurosurgery (1977-2023)

A total of 29 755 unique publications were published in *Neurosurgery* from 1977 to 2023, and bibliometric parameters were recorded (Table 2). Annual scholarly output ranged from 66 articles in 1977 and peaked at 1930 articles in 1998. Scientific output demonstrated a modest growth rate of 2.4%. Keyword analysis revealed subarachnoid hemorrhage, aneurysm, magnetic resonance imaging, meningioma, radiosurgery, and hydrocephalus as the most published terms since 1977. The seminal publications of *Neurosurgery*, a list of the 100 most cited articles throughout the journal's history, were recorded (Table 3).

**TABLE 2.** Bibliometric Evolution of *Neurosurgery* Publications From 1977 to 2023

Year	Total publications	Mean authorship count	% Small authorship groups (≤ 5 authors)	% Female first author	% Male first author	% Gender unknown first author	Mean # authorship affiliations	% Multinational authorship	% International authorship	% Database utilization (N)
1977	66	2.4	90.9	n.a.	n.a.	n.a.	1.33	0	6.7	n.a.
1978	128	2.6	96.9	n.a.	n.a.	n.a.	1.00	0	13.9	n.a.
1979	224	2.8	91.5	n.a.	n.a.	n.a.	1.01	0	12.9	n.a.
1980	252	2.8	96.4	n.a.	n.a.	n.a.	1.01	0.5	15.4	n.a.
1981	267	2.8	96.4	n.a.	n.a.	n.a.	1.01	0.4	18.5	n.a.
1982	329	3.2	89.4	n.a.	n.a.	n.a.	1.25	4.7	26.6	n.a.
1983	291	3.0	94.5	n.a.	n.a.	n.a.	1.01	0.4	27.8	n.a.
1984	309	3.2	91.6	n.a.	n.a.	n.a.	1.00	0	26.8	n.a.
1985	345	3.2	91.3	n.a.	n.a.	n.a.	1.01	0.3	32.5	n.a.
1986	340	3.5	90.0	n.a.	n.a.	n.a.	1.01	0.7	29.0	n.a.
1987	415	3.3	89.4	n.a.	n.a.	n.a.	1.08	1.5	37.8	n.a.
1988	387	3.6	85.3	n.a.	n.a.	n.a.	1.15	0.6	42.3	n.a.
1989	373	3.6	85.0	n.a.	n.a.	n.a.	1.01	0.9	38.8	n.a.
1990	415	3.5	85.8	7.5	78.8	13.7	1.01	0.6	44.4	n.a.
1991	413	3.5	82.3	4.0	83.1	12.9	1.00	0.3	47.4	n.a.
1992	458	3.4	86.2	5.3	73.5	21.2	1.91	5.9	43.3	n.a.
1993	481	3.2	87.1	3.8	78.4	17.9	1.78	7.1	36.2	n.a.
1994	482	3.4	83.0	6.8	81.1	12.1	1.92	5.6	37.7	n.a.
1995	504	3.4	85.3	4.8	84.3	10.9	1.84	6.6	33.0	n.a.
1996	739	2.9	87.4	4.5	84.5	11.0	3.01	9.8	38.1	n.a.
1997	849	3.0	86.6	4.9	80.5	14.6	3.05	9.0	49.1	n.a.
1998	1930	2.1	92.9	5.7	81.0	13.4	3.21	10.5	39.7	n.a.
1999	1434	2.4	90.4	4.3	81.3	14.4	3.09	8.8	38.8	n.a.
2000	895	3.1	83.0	4.3	83.6	12.1	2.59	9.8	39.0	n.a.
2001	871	3.1	83.7	5.0	79.1	15.9	2.99	9.5	39.7	n.a.
2002	709	3.0	84.9	6.6	78.0	15.4	2.76	10.8	43.2	n.a.
2003	657	3.0	83.7	7.7	69.2	23.1	2.94	12.8	50.0	n.a.
2004	769	3.5	78.8	8.4	72.1	19.6	3.24	10.1	38.5	n.a.
2005	815	3.8	74.4	7.0	71.7	21.3	3.14	16.8	39.6	n.a.
2006	777	4.2	69.2	6.6	71.0	22.4	3.45	14.4	38.1	n.a.
2007	787	3.5	78.0	7.9	73.6	18.5	3.42	14.9	41.4	0.38 (3)
2008	739	4.4	66.0	9.3	68.5	22.1	3.21	11.6	37.8	0.54 (4)
2009	593	4.6	64.1	9.8	68.0	22.2	3.08	17.6	38.0	0.34 (2)
2010	712	4.5	68.4	11.9	65.4	22.7	2.42	16.8	43.7	0.28 (2)
2011	639	4.5	65.7	10.5	73.0	16.5	3.03	16.1	44.1	0.78 (5)
2012	595	5.3	59.0	11.3	71.2	17.6	2.76	15.2	36.4	1.68 (10)
2013	621	5.0	61.0	10.8	72.2	17.0	3.17	20.4	34.8	1.61 (10)
2014	515	4.8	64.5	13.8	72.2	14.0	2.06	7.5	24.8	0.58 (3)
2015	555	5.1	60.9	11.6	70.7	17.7	2.27	8.8	19.6	1.62 (9)
2016	627	5.6	56.1	14.7	64.8	20.5	2.42	9.3	23.9	0.64 (4)
2017	688	5.4	58.3	13.0	68.7	18.4	2.37	8.3	24.9	1.31 (9)
2018	666	6.3	51.7	13.6	65.8	20.7	3.42	18.5	27.4	0.45 (3)
2019	1123	6.9	49.3	12.7	66.8	20.5	2.75	11.2	21.6	0.18 (2)
2020	1502	7.0	46.9	15.4	66.6	17.9	3.98	18.7	29.9	1.26 (19)
2021	769	8.0	43.4	16.1	67.2	16.7	4.07	21.8	27.6	1.82 (14)
2022	767	6.8	46.8	15.4	65.7	18.8	3.73	20.6	26.3	2.74 (21)
2023	933	8.4	37.1	15.7	63.7	20.6	4.60	21.2	29.6	2.14 (20)

n.a., not analyzed.

**TABLE 3.** The Seminal Publications of *Neurosurgery*

No.	Lead author	Date	Title	Total citation count^a^	Total citations per year^a^	Normalized Total Citations^a,b^
1	FISHER CM	1980	Relation of cerebral vasospasm to subarachnoid hemorrhage visualized by computerized tomographic scanning	2512	55.8	67.7
2	CARNEY N	2017	Guidelines for the Management of Severe Traumatic Brain Injury, Fourth Edition	2323	290.4	112.1
3	KNOSP E	1993	Pituitary adenomas with invasion of the cavernous sinus space: A magnetic resonance imaging classification compared with surgical findings	1124	35.1	23.7
4	MILHORAT TH	1999	Chiari I malformation redefined: Clinical and radiographic findings for 364 symptomatic patients	1026	39.5	16.7
5	SANAI N	2008	Glioma extent of resection and its impact on patient outcome	1014	59.6	71.6
6	GUSKIEWICZ KM	2005	Association between recurrent concussion and late-life cognitive impairment in retired professional football players	973	48.7	26.0
7	CIRIC I	1997	Complications of transsphenoidal surgery: Results of a national survey, review of the literature, and personal experience	928	33.1	13.2
8	GIZA CC	2014	The new neurometabolic cascade of concussion	900	81.8	65.3
9	RIMEL RW	1981	Disability caused by minor head injury	836	19.0	23.9
10	RELKIN N	2005	Diagnosing idiopathic normal-pressure hydrocephalus	775	38.8	20.7
11	OMALU BI	2005	Chronic traumatic encephalopathy in a National Football League player	772	38.6	20.7
12	LYLYK P	2009	Curative endovascular reconstruction of cerebral aneurysms with the pipeline embolization device: The Buenos Aires experience	712	44.5	57.0
13	CZOSNYKA M	1997	Continuous assessment of the cerebral vasomotor reactivity in head injury	702	25.1	10.0
14	DAUMAS-DUPORT C	1988	Dysembryoplastic neuroepithelial tumor: A surgically curable tumor of young patients with intractable partial seizures. Report of thirty-nine cases	701	18.9	17.4
15	BLACK PML	1997	Development and implementation of intraoperative magnetic resonance imaging and its neurosurgical applications	689	24.6	9.8
16	STUMMER W	2008	Extent of resection and survival in glioblastoma multiforme: Identification of and adjustment for bias	664	39.1	46.9
17	MAAS AIR	2005	Prediction of outcome in traumatic brain injury with computed tomographic characteristics: A comparison between the computed tomographic classification and combinations of computed tomographic predictors	661	33.1	17.7
18	DRAKE JM	1998	Randomized trial of cerebrospinal fluid shunt valve design in pediatric hydrocephalus	629	23.3	11.9
19	ALBERT FK	1994	Early postoperative magnetic resonance imaging after resection of malignant glioma: Objective evaluation of residual tumor and its influence on regrowth and prognosis	620	20.0	13.6
20	STUMMER W	1998	Intraoperative detection of malignant gliomas by 5-aminolevulinic acid- induced porphyrin fluorescence	615	22.8	11.6
21	GILLER CA	1993	Cerebral arterial diameters during changes in blood pressure and carbon dioxide during craniotomy	600	18.8	12.6
22	RAABE A	2003	Near-infrared indocyanine green video angiography: A new method for intraoperative assessment of vascular flow	595	27.0	10.1
23	WILLIAMS DH	1990	Mild head injury classification	587	16.8	13.2
24	NORTH RB	2005	Spinal cord stimulation versus repeated lumbosacral spine surgery for chronic pain: A randomized, controlled trial	584	29.2	15.6
25	PELLMAN EJ	2003	Neurosurgical outcomes in a modern series of 400 craniotomies for treatment of parenchymal tumors	564	25.6	9.6
26	SAWAYA R	1998	Concussion in professional football: Reconstruction of game impacts and injuries	564	20.9	10.6
27	KASSAM AB	2008	Endoscopic reconstruction of the cranial base using a pedicled nasoseptal flap	563	33.1	39.8
28	SAMII M	1997	Management of 1000 vestibular schwannomas (acoustic neuromas): Surgical management and results with an emphasis on complications and how to avoid them	550	19.6	7.8
29	KROLL RA	1998	Outwitting the blood-brain barrier for therapeutic purposes: Osmotic opening and other means	540	20.0	10.2
30	KASSELL NF	1982	Treatment of ischemic deficits from vasospasm with intravascular volume expansion and induced arterial hypertension	528	12.3	17.7
31	GIESE A	1996	Glioma invasion in the central nervous system	523	18.0	7.7
32	PANG D	1992	Split cord malformation: Part I: A unified theory of embryogenesis for double spinal cord malformations	516	15.6	12.4
33	BOUTHILLIER A	1996	Segments of the internal carotid artery: A new classification	502	17.3	7.4
34	FRONTERA JA	2006	Prediction of symptomatic vasospasm after subarachnoid hemorrhage: The modified fisher scale	495	26.1	21.5
35	GOEL A	2002	Atlantoaxial fixation using plate and screw method: A report of 160 treated patients	479	20.8	8.2
36	LOZIER AP	2002	Ventriculostomy-related infections: A critical review of the literature	479	20.8	8.2
37	MCDANNOLD N	2010	Transcranial magnetic resonance imaging- guided focused ultrasound surgery of brain tumors: Initial findings in 3 patients	478	31.9	28.3
38	TANIGUCHI M	1993	Modification of cortical stimulation for motor evoked potentials under general anesthesia: Technical description	476	14.9	10.0
39	BARKER AT	1987	Magnetic stimulation of the human brain and peripheral nervous system: an introduction and the results of an initial clinical evaluation.	476	12.5	12.2
40	POLIN RS	1997	Decompressive bifrontal craniectomy in the treatment of severe refractory posttraumatic cerebral edema	468	16.7	6.7
41	NIMSKY C	2000	Quantification of, visualization of, and compensation for brain shift using intraoperative magnetic resonance imaging	466	18.6	7.2
42	NORTH RB	1993	Spinal cord stimulation for chronic, intractable pain: Experience over two decades	463	14.5	9.8
43	AL-MEFTY O	1988	Petrosal approach for petroclival meningiomas	454	12.3	11.3
44	KANPOLAT Y	2001	Percutaneous controlled radiofrequency trigeminal rhizotomy for the treatment of idiopathic trigeminal neuralgia: 25-year experience with 1600 patients	453	18.9	7.8
45	CLAUS EB	2005	Epidemiology of intracranial meningioma	445	22.3	11.9
46	KAWASE T	1991	Anterior transpetrosal-transtentorial approach for sphenopetroclival meningiomas: Surgical method and results in 10 patients	437	12.9	10.5
47	HLAVIN ML	1990	Spinal epidural abscess: A ten-year perspective	437	12.5	9.8
48	CARDINALE F	2013	Stereoelectroencephalography: Surgical methodology, safety, and stereotactic application accuracy in 500 procedures	437	36.4	22.4
49	RACINE R	1978	Kindling: the first decade	435	9.3	16.6
50	KUMAR K	2008	Physiological basis of motor effects of a transient stimulus to cerebral cortex	430	25.3	30.4
51	AMASSIAN VE	1987	The effects of spinal cord stimulation in neuropathic pain are sustained: A 24-month follow-up of the prospective randomized controlled multicenter trial of the effectiveness of spinal cord stimulation	430	11.3	11.0
52	SIEGFRIED J	1994	Bilateral chronic electrostimulation of ventroposterolateral pallidum: A new therapeutic approach for alleviating all parkinsonian symptoms	428	13.8	9.4
53	DHAR S	2008	Morphology parameters for intracranial aneurysm rupture risk assessment	424	24.9	30.0
54	HOPF NJ	1999	Endoscopic third ventriculostomy: Outcome analysis of 100 consecutive procedures	422	16.2	6.9
55	MIZUTANI T	1995	Recurrent subarachnoid hemorrhage from untreated ruptured vertebrobasilar dissecting aneurysms	421	14.0	9.0
56	COULDWELL WT	2004	Variations on the standard transsphenoidal approach to the sellar region, with emphasis on the extended approaches and parasellar approaches: Surgical experience in 105 cases	419	20.0	9.3
57	NABAVI A	2001	Management of 1000 vestibular schwannomas (acoustic neuromas): Clinical presentation	416	17.3	7.1
58	MATTHIES C	1997	Serial intraoperative magnetic resonance imaging of brain shift	415	14.8	5.9
59	HAGLUND MM	1994	Cortical localization of temporal lobe language sites in patients with gliomas	411	13.3	9.0
60	ROBERTS DW	1998	Intraoperative brain shift and deformation: A quantitative analysis of cortical displacement in 28 cases	410	15.2	7.7
61	SAMII M	1997	Management of 1000 vestibular schwannomas (acoustic neuromas): Hearing function in 1000 tumor resections	406	14.5	5.8
62	MCGIRT MJ	2008	Effect of the extent of surgical resection on survival and quality of life in patients with supratentorial glioblastomas and anaplastic astrocytomas	405	23.8	28.6
63	AMMIRATI M	1987	Extent of surgical resection is independently associated with survival in patients with hemispheric infiltrating low-grade gliomas	405	10.7	10.4
64	HERNESNIEMI JA	2008	Natural history of brain arteriovenous malformations: A long-term follow-up study of risk of hemorrhage in 238 patients	402	23.6	28.4
65	MORITA A	1993	Esthesioneuroblastoma: Prognosis and management	401	12.5	8.5
66	DUMONT AS	2003	Cerebral vasospasm after subarachnoid hemorrhage: Putative role of inflammation	397	18.0	6.7
67	OH MY	2002	Long-term hardware-related complications of deep brain stimulation	396	17.2	6.8
68	SIMARD JM	1986	Cavernous angioma: A review of 126 collected and 12 new clinical cases	396	10.2	12.1
69	TANG JA	2012	The impact of standing regional cervical sagittal alignment on outcomes in posterior cervical fusion surgery	396	30.5	22.7
70	HEBB AO	2001	Idiopathic normal pressure hydrocephalus: A systematic review of diagnosis and outcome	387	16.1	6.6
71	SAMII M	1997	Management of 1000 vestibular schwannomas (acoustic neuromas): The facial nerve - Preservation and restitution of function	386	13.8	5.5
72	MAAS AIR	2015	Collaborative European neurotrauma effectiveness research in traumatic brain injury (CENTER-TBI): A prospective longitudinal observational study	386	38.6	25.9
73	ROSS JS	1996	Association between Peridural Scar and Recurrent Radicular Pain after Lumbar Discectomy: Magnetic Resonance Evaluation	385	13.3	5.7
74	GAY E	1995	Chordomas and chondrosarcomas of the cranial base: Results and follow-up of 60 patients	385	12.8	8.2
75	NUTTIN BJ	2003	Microsurgical anatomy of the posterior inferior cerebellar artery	381	17.3	6.5
76	LISTER JR	1982	Long-term electrical capsular stimulation in patients with obsessive-compulsive disorder	381	8.9	12.8
77	AGHI MK	2009	Long-term recurrence rates of atypical meningiomas after gross total resection with or without postoperative adjuvant radiation	380	23.8	30.4
78	VALTONEN S	1997	Interstitial chemotherapy with carmustine-loaded polymers for high- grade gliomas: A randomized double-blind study	378	13.5	5.4
79	HENDERSON CM	1983	Posterior-lateral foraminotomy as an exclusive operative technique for cervical radiculopathy: A review of 846 consecutively operated cases	378	9.0	11.6
80	WALTERS BC	2013	Guidelines for the management of acute cervical spine and spinal cord injuries: 2013 update	378	31.5	19.4
81	GUTHRIE BL	1989	Meningeal hemangiopericytoma: Histopathological features, treatment, and long-term follow-up of 44 cases	377	10.5	8.6
82	LAWTON MT	2010	A supplementary grading scale for selecting patients with brain arteriovenous malformations for surgery	377	25.1	22.3
83	KASSAM AB	2005	The expanded endonasal approach: A fully endoscopic transnasal approach and resection of the odontoid process: Technical case report	376	18.8	10.1
84	HAINES DE	1993	The “subdural” space: A new look at an outdated concept	375	11.7	7.9
85	GREENWALD RM	2008	Head impact severity measures for evaluating mild traumatic brain injury risk exposure	371	21.8	26.2
86	VAN DEN BRINK WA	2000	Brain oxygen tension in severe head injury	371	14.8	5.7
87	ADLER JR. JR	1999	Image-guided robotic radiosurgery	371	14.3	6.0
88	OMALU BI	2006	Chronic traumatic encephalopathy in a National Football League player: Part II	370	19.5	16.1
89	SEN CN	1990	An extreme lateral approach to intradural lesions of the cervical spine and foramen magnum	369	10.5	8.3
90	BERGER MS	1989	Brain mapping techniques to maximize resection, safety, and seizure control in children with brain tumors	368	10.2	8.4
91	ROVLIAS A	2000	The influence of hyperglycemia on neurological outcome in patients with severe head injury	366	14.6	5.7
92	HILL DLG	1998	Measurement of intraoperative brain surface deformation under a craniotomy	366	13.6	6.9
93	UJIIE H	1999	Effects of size and shape (aspect ratio) on the hemodynamics of saccular aneurysms: A possible index for surgical treatment of intracranial aneurysms	365	14.0	6.0
94	MACIUNAS RJ	1994	The application accuracy of stereotactic frames	362	11.7	8.0
95	BILLS DC	1993	A retrospective analysis of pituitary apoplexy	362	11.3	7.6
96	UJIIE H	2001	Is the aspect ratio a reliable index for predicting the rupture of a saccular aneurysm?	358	14.9	6.1
97	GARDNER PA	2008	Endoscopic endonasal resection of anterior cranial base meningiomas	357	21.0	25.2
98	STAFFORD SL	2001	Meningioma radiosurgery: Tumor control, outcomes, and complications among 190 consecutive patients	357	14.9	6.1
99	IKEDA Y	1990	The molecular basis of brain injury and brain edema: The role of oxygen free radicals	357	10.2	8.0
100	DEHDASHTI AR	2008	Pure endoscopic endonasal approach for pituitary adenomas: Early surgical results in 200 patients and comparison with previous microsurgical series	356	20.9	25.2

a As of July 2024.

b Normalized Total Citations = total number of citations/average number of citations of all documents published in same year.

The 100 most cited publications in *Neurosurgery* through its modern history (1977-2023).

### Total Authorship Count

**FIGURE 1.**
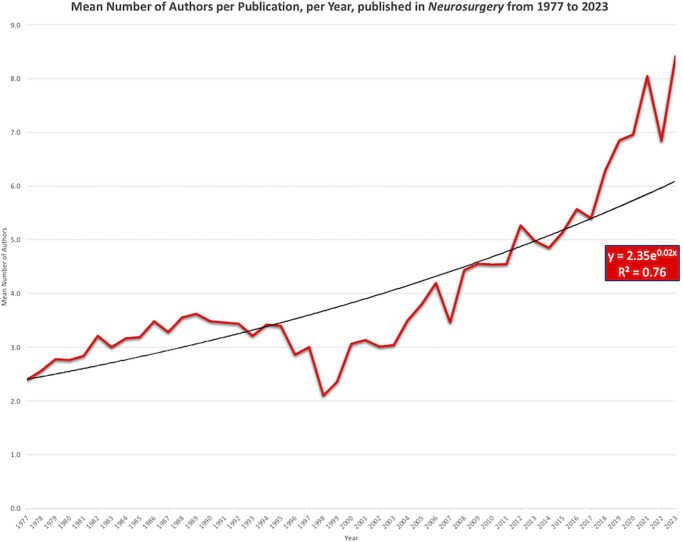
The mean number of authors per publication in *Neurosurgery* has demonstrated exponential growth at 2.0% annually from 1977 to 2023. The mean authorship count was lowest in 1998 (2.1 authors) and highest in 2023 (8.4 authors).

**FIGURE 2.**
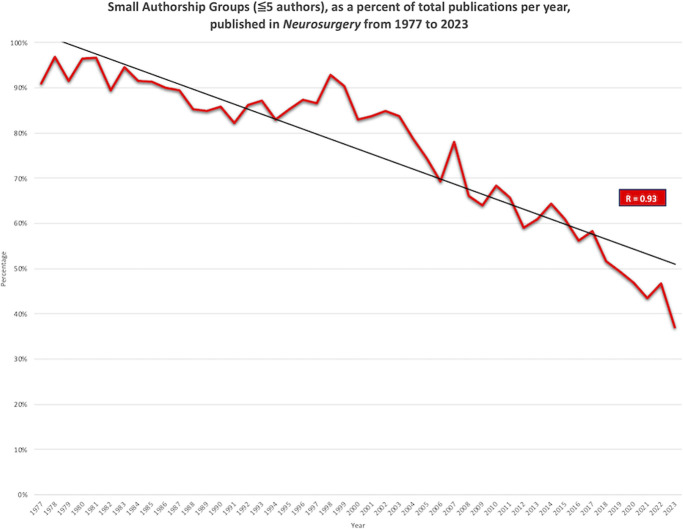
The percentage of publications with small authorship groups (≤ 5 authors) in *Neurosurgery* has demonstrated a strong linear decrease from 1977 (91% of publications) to 2023 (37% of publications) (*R* = 0.94).

The mean number of authors, per year, demonstrated exponential growth from 1977 (2.4) to 2023 (8.4) (*P* < .001) (Figure 1). The exponential model demonstrated a 2.0% annual growth rate in total author number with a strong coefficient of determination (*R*^2^ = 0.76). The percentage of publications, per year, with a small authorship group (≤ 5 authors) linearly decreased from 1977 (91%) to 2023 (37%) (*R* = 0.93) (*P* < .001) (Figure 2).

### First Author Gender Representation

**FIGURE 3.**
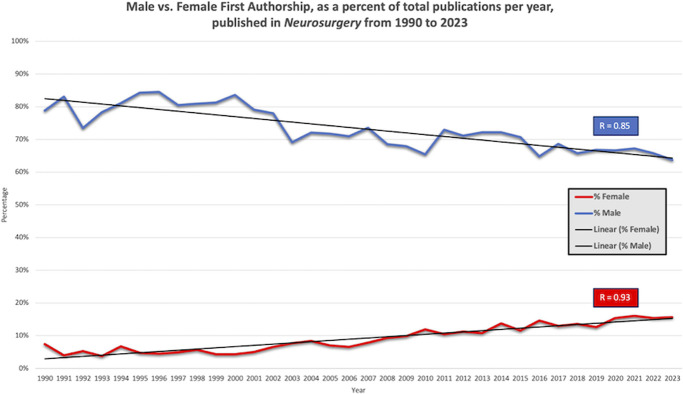
Female first authorship in *Neurosurgery* has demonstrated a strong linear increase from 1990 (7%) to 2023 (16%), with the lowest rate in 1993 (4%) (*R* = 0.93). Male first authorship has seen a linear decrease from 1990 (79%) to 2023 (64%), with the highest rate in 1996 (85%) (*R* = 0.85). 17% of publications had a first author with an ambiguous or unspecified sex, and were excluded from this analysis.

Percentage of publications, per year, with female first authorship demonstrated a strong linear increase from 1990 (7.5%) to 2023 (15.7%) (*R* = 0.93) (*P* < .001) (Figure 3). The percentage of publications, per year, with male first authorship demonstrated a strong linear decrease from 1990 (78.8%) to 2023 (63.7%) (*R* = 0.85) (*P* < .001) (Figure 3). The mean percentage of authors with an unspecific gender was 17%, whom were excluded from the analysis to avoid gender misrepresentation.

### First and Senior Authorship Degree Representation

**FIGURE 4.**
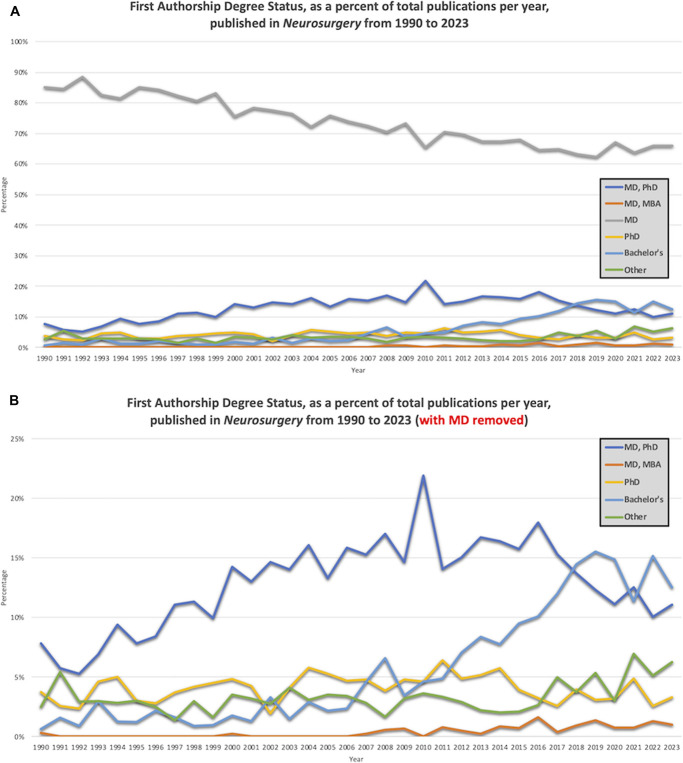
The degree status of first authors published in *Neurosurgery* from 1990 to 2023. **A**, The percentage of publications with an MD-only first author decreased from 1990 (85%) to 2023 (65.8%), with the lowest rate in 2019 (62.4%). **B**, Over the same time period, the percentage of publications first authored by a Bachelor degree increased (1990: 0.6%, 2023: 12.5%), with the highest rate in 2019 (15.5%).

Degree status among first authors published in *Neurosurgery* varied significantly from 1990 to 2023 (Figure 4). MD-only first authorship decreased over the study period from 85.0% (1990) to 65.8% (2023) (*P* < .001). Over the same time period, the percentage of publications first authored by a Bachelor degree increased from 0.6% (1990) to 12.5% (2023) (*P* < .001).

**FIGURE 5.**
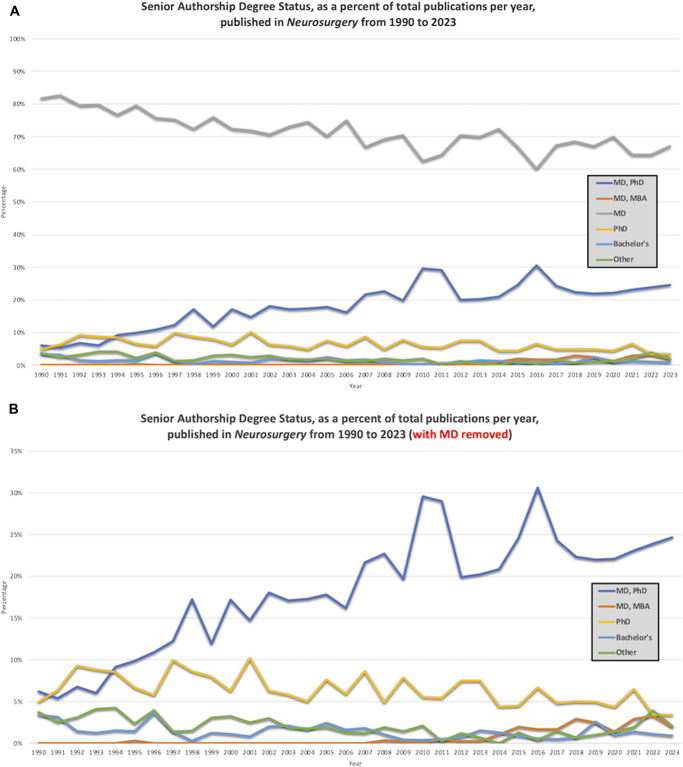
The degree status of senior authors published in *Neurosurgery* from 1990 to 2023. **A**, MD-only senior authorship declined over the study period (1990: 81.8%, 2023: 67.0%). **B**, The percentage of publications with an MD/PhD senior author increased from 1990 (6.2%) to 2023 (24.7%), with the highest rate in 2016 (30.6%). Over the same time period, the percentage of publications senior authored by a MD/MBA degree increased (1990: 0%, 2023: 1.9%), with the highest rate in 2022 (3.3%).

Degree status among senior authors published in *Neurosurgery* varied significantly from 1990 to 2023 (Figure 5). MD-only senior authorship decreased over the study period from 81.8% (1990) to 67.0% (2023) (*P* < .001). Over the same time period, the percentage of senior author publications by an MD/PhD increased from 6.2% (1990) to 24.7% (2023) (*P* < .001). The percentage of publications senior authored by an MD/MBA also increased from 1990 (0%) to 2023 (1.9%) (*P* < .001).

### Authorship Collaboration in Neurosurgery

Multinational authorship groups linearly increased in *Neurosurgery* from 1977 (0%) to 2023 (21.2%) (*R* = 0.95) (*P* < .001) (Figure 6a). The mean number of authorship affiliations per publication in *Neurosurgery* increased exponentially at a 3.2% annual rate from 1977 (1.3) to 2023 (4.6) (*R*^2^ = 0.65) (*P* < .001) (Figure 6b). International authorship groups steadily increased from 1977 (6.7%) and peaked in 2003 (50.0%), but have since declined to 29.6% in 2023 (Figure 6c).

FIGURE 6.Authorship collaboration has increased in *Neurosurgery* from 1977 to 2023. **A**, The percentage of publications with more than one authorship nationality increased linearly from 1977 (0%) to 2023 (21.2%) (*R* = 0.95) (*P* < .001). **B**, The mean number of authorship affiliations per publication increased exponentially at a 3.2% annual rate from 1977 (1.3) to 2023 (4.6) (*R*^2^ = 0.65) (*P* < .001). **C**, International authorship groups steadily increased from 1977 (6.7%), peaked in 2003 (50.0%), and have declined to 29.6% in 2023.

**Figure:**
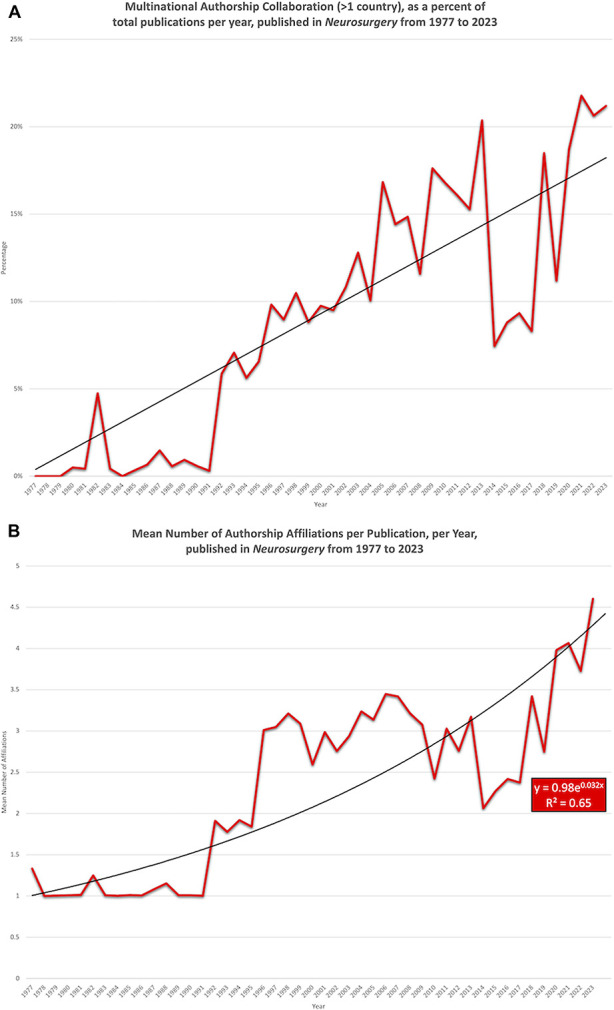

**Figure:**
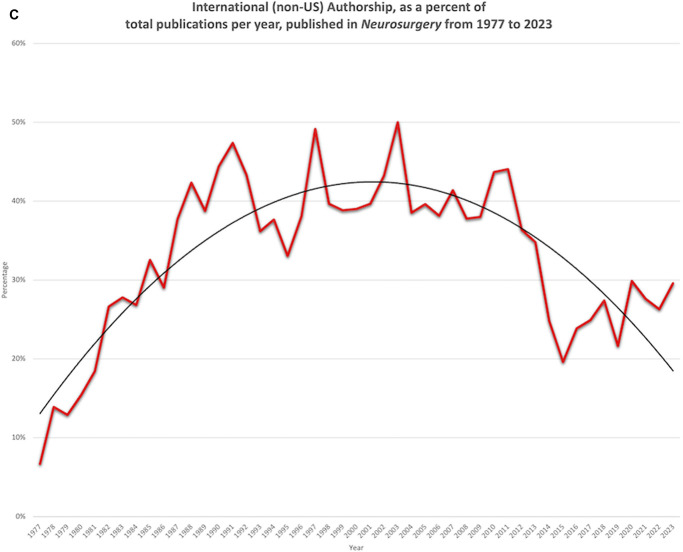

### Journal Content, Publication Type, and Database Utilization in Neurosurgery

**FIGURE 7.**
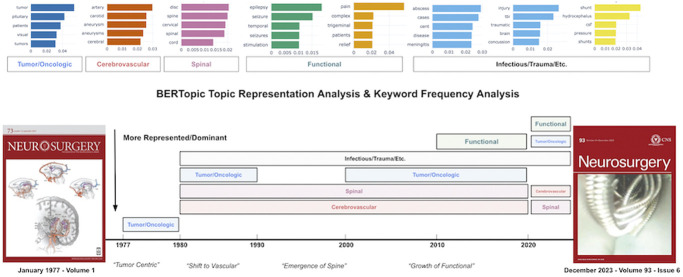
Representative BERTopic-generated theme categories and their evolution in Neurosurgery from 1977 to 2023. Tumor topics were most represented in the 1970s, followed by cerebrovascular topics from the 1980s to 2010s and, more recently, spinal topics in the 2020s. Images reprinted with permission. Neurosurgery, volume 1, issue 2, https://journals.lww.com/neurosurgery/toc/1977/07000; Neurosurgery, volume 93, issue 6, https://journals.lww.com/neurosurgery/toc/2023/12000. BERT, Bidirectional Encoder Representations from Transformers.

**FIGURE 8.**
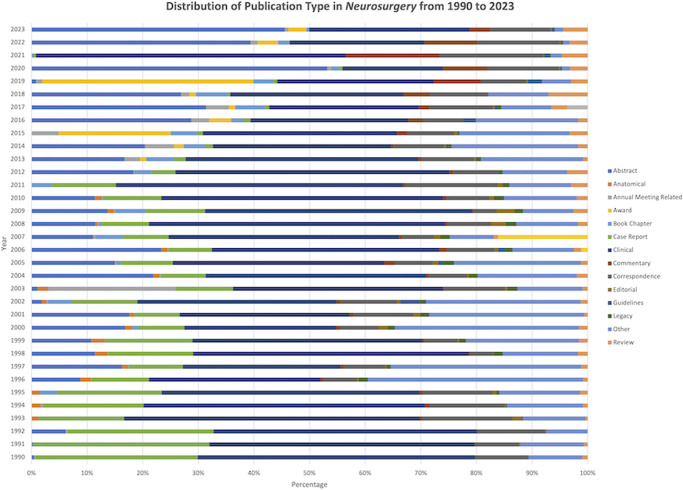
Distribution of publication type in *Neurosurgery* from 1990 to 2023. Case reports have declined in prevalence, clinical articles have remained stable, and reviews have increased in prevalence from 1990 to 2023.

**FIGURE 9.**
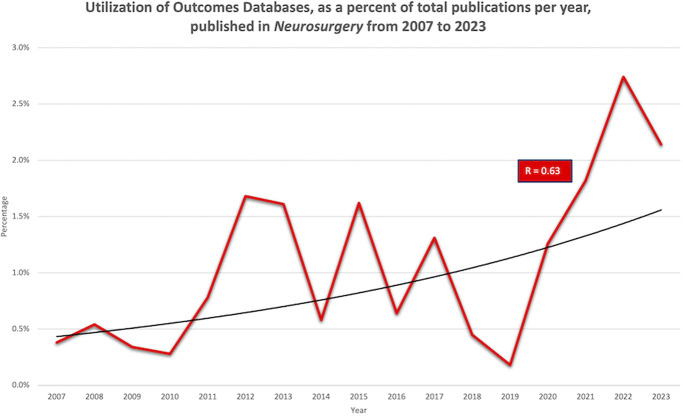
The utilization of outcomes databases in *Neurosurgery* from 2007 to 2023. The percentage of publications that used an outcomes database in *Neurosurgery* has demonstrated an exponential increase from 2007 (0.38%) to 2023 (2.14%), with the highest rate in 2022 (2.74%) (*R* = 0.63).

Keyword frequency analysis and BERTopic modeling consistently identified dominant themes in *Neurosurgery* for each decade. The 1970s were predominantly focused on tumors, shifting toward cerebrovascular topics in the 1980s which have remained dominant through the 2010s. Spinal themes emerged in the 1980s and have overtaken cerebrovascular as the most represented subspecialty in *Neurosurgery* in the 2020s. The 2010s saw the rise of functional neurosurgery topics, including epilepsy and deep brain stimulation (Figure 7). Publication types in *Neurosurgery* have demonstrated heterogeneity since 1990 (Figure 8). Case reports have declined in prevalence, clinical articles have remained stable, and reviews have increased in prevalence from 1990 to 2023. A total of 140 outcomes database studies were published in *Neurosurgery* from 2007 to 2023. Database utilization, as a percentage of total publications per year, demonstrated an exponential, 4.6-fold increase from 2007 (0.38%) to 2023 (2.14%) (*R* = 0.63) (*P* < .001) (Figure 9).

## DISCUSSION

This bibliometric analysis of *Neurosurgery* publications demonstrated increasing authorship counts and decreased rates of small authorship groups from 1977 to 2023. From 1990 to 2023, the journal experienced increased first authorship among female authors and authors with Bachelor degrees, increased MD/PhD senior authorship, and a decrease in MD-only first and senior authorship. The journal's content has shifted toward topics in spine and cerebrovascular neurosurgery with more review articles and less case reports. From 2007 to 2023, studies in *Neurosurgery* that analyzed outcomes databases increased 4.6-fold. The evolution of *Neurosurgery*'s journal content reflects the increased collaboration, authorship diversity, and big data utilization that have characterized neurosurgical publishing over the past several decades.^16-18^

### Increasing Authorship: Collaboration or Courtesy?

Few studies have evaluated collaboration and increased authorship counts in neurosurgical research.^10,19-21^ Cole et al^10^ demonstrated increased authorship number across the *Journal of Neurosurgery* journals and a ∼20% increase in publications in *Neurosurgery* with 10 or more authors over the past 40 years. The COVID-19 pandemic catalyzed a seismic shift in the way research is conducted, leading to remote avenues to cross-disciplinary and multi-institutional collaboration.^10,22^ In *Neurosurgery*, 11.8% of publications had multinational authorship groups in 2019, nearly doubling to 21.8% in 2021, correlating with the COVID-19 pandemic. Alternatively, the exponential increase in authorship count may be attributed to increased research complexity, larger research teams, or increased funding resources.^10,20,23^

Honorary authorship, a concept that has received increasing attention across academic publishing, may be another driver of an increasing authorship count. Gifting authorship may have multiple motivations, including strengthening professional relationships and research networks. There is a common belief that including senior authors with tested academic reputations will increase the chances of publication in a top-tier journal.^23^ *Neurosurgery* blinds peer reviewers to authors to avoid bias in evaluating scientific research, which is not a universal practice. Cole et al^10^ demonstrated that only 28% of increased authorship counts since 1980 was due to increased multidepartmental or multi-institutional authorship. Although these findings may indicate increased intradepartmental collaboration, they may also suggest a greater prevalence of honorary authorship for colleagues within one's department. Guest authorship is a particularly concerning explanation for increased author counts because it undermines the integrity of authorship contributions. With the proliferation of reviews and database utilization seen in this study, it is presumably easier to publish large patient series than it once was when large teams were required for “chart mining.” Thus, there is no clear reason why authorship count should be increasing exponentially since the journal's inception. Although *Neurosurgery* has promoted authorship accountability with required authorship contribution statements for each submission, it remains difficult to transparently assess authorship contributions. It is understandable why first authors without tenure are more likely to have guest authors compared with publications with associate or full professors listed as first authors.^24^ There is an assumption that multiauthor and multi-institutional studies exemplify greater expertise and may have a greater impact on the field.^11^ However, an increase in authorship count is not a significant predictor of increased citation count.^20^ Further study is necessary to identify the behavioral factors that have led to this widespread publishing trend in neurosurgery and academia.

### Promoting Female Authorship in Neurosurgery

Female first authorship in *Neurosurgery* has demonstrated strong linear growth since 1990. The 72% gender gap in first authorship in 1990 has been narrowed to 48% in 2023. Although these findings indicate improvement, there is significant work that remains to address well-established gender disparities in neurosurgery.^25^ The Women in Neurosurgery white paper was published 15 years ago to address recruitment and retention of female neurosurgical residents and practitioners, and female first authorship has increased in *Neurosurgery* since.^26^ However, a 2019 study revealed women constitute only 12% of academic neurosurgeons in the United States and Canada, while modest increases have been made in the number of female neurosurgery residents.^27-30^ In 2019, the female first authorship representation in *Neurosurgery* was 13% of all publications, consistent with the active female neurosurgery workforce. Female representation in the journal is expected to continue growing, as nearly 30% of neurosurgery residents are female.^31^ Concerted efforts have been made to evaluate barriers for female medical students, who are less likely to match successfully in neurosurgery.^32^ Given the neurosurgery publication “arms race” among medical students, there may be a correlation between decreased female scholarly representation in *Neurosurgery* and their decreased likelihood of matching.^33^ For early-career neurosurgeons, there is an emphasis on publishing for academic career advancement; however, women are also underrepresented as senior authors.^34^ In addition, female neurosurgeons have significantly higher attrition rates, which have been attributed to suboptimal mentorship, home expectations, and workplace gender bias.^35^ Although the present data demonstrates encouraging growth in female first authorship, future studies may determine direct relationships between female scholarly output and causal factors.^36^

### The Rise of Medical Student Authorship in Neurosurgery

A significant increase in medical student scholarly output has occurred over the past two decades. The average neurosurgery applicant had 1.7 indexed publications in 2009, increasing to 8.1 in 2020.^33,37^ The present findings suggest increased medical student authorship in *Neurosurgery* because the percentage of publications with a medical student first author increased from 0.6% (1990) to 12.5% (2023). Medical student scholarly productivity has emerged as a leading predictor of neurosurgery “Match” success. However, disparities in research opportunities are evident because men, international medical graduates, and students from top 40 medical schools are significantly more likely to have increased publication counts.^38^ Top-ranked medical schools are more likely to have well-established research infrastructure and faculty networks from which students may benefit.^39^ The publication “arms race” has also revealed an increase in student scholarly misrepresentation.^40^ From 2006 to 2012, applicant scholarly misrepresentation increased from 33% to 45% and was more prevalent among students with a greater number of reported works or from unranked medical schools.^40^ *Neurosurgery's* leadership example of publishing medical student first authors is a testament to the journal's rigorous peer-review process, as blinded reviewers may have bias toward medical student submissions. Future studies may investigate the increase in medical student involvement in *Neurosurgery,* as publishing culture ideally shifts to less quantity and more quality among medical student scholarly work.

### Database Utilization in Neurosurgery

Outcomes database utilization in *Neurosurgery* has seen an exponential, 4.6-fold increase from 2007 to 2023. These findings may be a product of the 2009 Institute of Medicine and the American Recovery and Reinvestment Act, which called for the development of prospective registries to capture patient-centered data from current practice to inform evidence-based care.^41^ The rate of outcomes database use in major neurosurgery journals has increased from ∼0.5% in 2005 to exceeding 3.0% in 2020.^42^ In 2020, the highest rate of registry use was in *Neurosurgical Focus*.^42^ Outcomes databases offer unprecedented power, which while a strength, may also result in statistical significance that may be a product of sheer sample size.^42^ “Database mining” may also be responsible for the proliferation of scientific output, potentially underscoring the nearly 4-fold increase in scientific “super researchers” who publish more than 60 papers per year.^43^ The rampant increase in total authorship count and database utilization may be beheld with skepticism because database studies require significantly less personnel than traditional chart review. Similarly, future work may investigate the intersection between database utilization among medical student authors as it relates to the publication “arms race.”

### Limitations

This study has several limitations inherent to its design. The sample size and ambiguity of the *gender_guesser* Python package resulted in 17% of authors being excluded from the gender representation analysis, which may result in suboptimal accuracy. However, gender_guesser achieved the lowest rate of gender misclassifications at 2.6% compared with other statistical packages, supporting its use in this study.^13^ In addition, the database utilization analysis may have missed nuanced, less commonly used databases published in *Neurosurgery*. For the affiliations analysis, increased authorship affiliation, while it sought to identify cross-institutional affiliation, may have captured authors with multiple affiliations at one institution, potentially inflating these results. Last, Bachelor degree first authorship served as a surrogate for medical student authorship, while college students and international medical graduates applying to residency would not have been captured with this degree tag.

## CONCLUSION

*Neurosurgery* publications have experienced a bibliometric evolution from 1977 to 2023. The journal has a nearly 4-fold increase in authorship number, a sharp decline in small authorship groups (≤ 5 authors), increased female and medical student first authorship, and increased MD/PhD senior authorship. Authorship collaboration has blossomed among institutions and internationally since the journal's inception in 1977. Topics in cerebrovascular and spine research are most represented, with an increase in review articles and decrease in case reports in recent years. Outcomes database utilization in the journal has also increased 4.6-fold from 2007 to 2023. Future studies may collect more granular, prospective data of each publication in *Neurosurgery* for analysis.

## References

[1] Wilkins RH. Birth of a journal: the origin and early years of Neurosurgery. Neurosurgery. 1982;10(6 Pt 2):820-826. 7050760 10.1227/00006123-198206020-00001

[2] Kurland DB Save A Patel A, et al. The evolution of skull base surgery: a bibliometric analysis spanning nearly 125 years. J Neurol Surg B Skull Base. Published online July 22, 2024. doi: 10.1055/s-0044-1788636 PMC12227253 40620625

[3] Mir JM Kurland DB Cheung ATM The evolution of pediatric spine surgery: a bibliometric analysis of publications from 1902 to 2023. Neurosurg Pract. 2024;5(3):e00092. 10.1227/neuprac.0000000000000092 PMC11783662 39959902

[4] Kurland DB Cheung ATM Kim NC A century of evolution in spine surgery publications: a bibliometric analysis of the field from 1900 to 2023. Neurosurgery. 2023;93(5):1121-1143. 37610208 10.1227/neu.0000000000002648

[5] Geng Y Xie L Li J Wang Y Li X. Bibliometric analysis of emerging trends and research foci in brainstem tumor field over 30 years (1992-2023). Childs Nerv Syst. 2024;40(6):1901-1917. 38630267 10.1007/s00381-024-06404-w

[6] Garg K Chaurasia B Gienapp AJ Splavski B Arnautovic KI. Bibliometric analysis of publications from 2011-2020 in 6 major neurosurgical journals (Part 1): geographic, demographic, and article type trends. World Neurosurg. 2022;157:125-134. 34753011 10.1016/j.wneu.2021.10.091

[7] Niquen-Jimenez M Wishart D Garcia RM A Bibliographic analysis of the most cited articles in global neurosurgery. World Neurosurg. 2020;144:e195-e203. 32829020 10.1016/j.wneu.2020.08.084 PMC7895493

[8] Kanmounye US Robertson FC Sebopelo LA Bibliometric analysis of the 200 most cited articles in WORLD NEUROSURGERY. World Neurosurg. 2021;149:226-231.e3. 33548539 10.1016/j.wneu.2021.01.121

[9] Karhade AV Larsen AMG Cote DJ Dubois HM Smith TR. National databases for neurosurgical outcomes research: options, strengths, and limitations. Neurosurgery. 2018;83(3):333-344. 28950367 10.1093/neuros/nyx408

[10] Cole TS Pacult MA Lawton MT. Increasing author counts in neurosurgical journals from 1980 to 2020. J Neurosurg. 2022;136(2):584-588. 34359040 10.3171/2021.1.JNS204257

[11] Harsh D Adnan HS Raees AP Manjul T Anil N. How many neurosurgeons does it take to author an article and what are the other factors that impact citations? World Neurosurg. 2021;146:e993-e1002. 33220479 10.1016/j.wneu.2020.11.058

[12] Pérez IS. gender-guesser: Get the gender from first name. Accessed July 15, 2024. https://github.com/lead-ratings/gender-guesser

[13] Santamaría L Mihaljević H. Comparison and benchmark of name-to-gender inference services. PeerJ Comput Sci. 2018;4:e156. 10.7717/peerj-cs.156 PMC7924484 33816809

[14] Aria M Cuccurullo C. *bibliometrix*: An R-tool for comprehensive science mapping analysis. J Informetrics. 2017;11(4):959-975.

[15] Kurland DB Lau D Kim NC Ames C. A bibliometric analysis of patient-reported outcome measures in adult spinal deformity, and the future of patient-centric outcome assessments in the era of predictive analytics. Semin Spine Surg. 2023;35(2):101032.

[16] Jelmoni AJ Cannizzaro D Uralov D Collaborative initiatives in neurosurgery research and publications between high-income and low/middle-income countries: a bibliometric analysis. Neurosurgery. 2024;95(4):e121-e131. 39283118 10.1227/neu.0000000000002935 PMC11377094

[17] Farhan SA Shahid I Siddiqi J Khosa F. Assessing the gap in female authorship in neurosurgery literature: a 20-year analysis of sex trends in authorship. World Neurosurg. 2020;141:e661-e669. 32522642 10.1016/j.wneu.2020.05.248

[18] Raju B Jumah F Ashraf O Big data, machine learning, and artificial intelligence: a field guide for neurosurgeons. J Neurosurg. 2021;135(2):373-383. 33007750 10.3171/2020.5.JNS201288

[19] Frey CD Wilson TA Decamillis M A pilot study of the level of evidence and collaboration in published neurosurgical research. World Neurosurg. 2017;108:901-908. 28899833 10.1016/j.wneu.2017.09.011

[20] King JT. How many neurosurgeons does it take to write a research article? Authorship proliferation in neurosurgical research. Neurosurgery. 2000;47(2):435-440. 10942017 10.1097/00006123-200008000-00032

[21] Eremiev A Kurland DB Carter C Trends in the corpus of literature on endoscopic third ventriculostomy: a bibliometric analysis spanning 3 decades. J Neurosurg Pediatr. 2024;34(4):334-346. 39059455 10.3171/2024.5.PEDS24135

[22] Tosi U Chidambaram S Schwarz J The world of neurosurgery reimagined post COVID-19: crisis ↔ opportunities. World Neurosurg. 2021;148:251-255. 33770847 10.1016/j.wneu.2020.11.167 PMC9760480

[23] Tilak G Prasad V Jena AB. Authorship inflation in medical publications. Inquiry. 2015;52:0046958015598311. 26228035 10.1177/0046958015598311 PMC4943864

[24] Slone RM. Coauthors’ contributions to major papers published in the AJR: frequency of undeserved coauthorship. AJR Am J Roentgenol. 1996;167(3):571-579. 8751654 10.2214/ajr.167.3.8751654

[25] Abosch A Rutka JT. Women in neurosurgery: inequality redux. J Neurosurg. 2018;129(2):277-281. 29999441 10.3171/2018.4.JNS172878

[26] WINS White Paper Committee, Benzil DL Abosch A Germano I The future of neurosurgery: a white paper on the recruitment and retention of women in neurosurgery. J Neurosurg. 2008;109(3):378-386. 18759565 10.3171/JNS/2008/109/9/0378

[27] Odell T Toor H Takayanagi A Gender disparity in academic neurosurgery. Cureus. 2019;11(5):e4628. 31312554 10.7759/cureus.4628 PMC6623992

[28] Smith EC Calafiore RL Oravec CS Kittel C Wolfe SQ White JJ. Fueling the future of neurosurgery: increasing trends in female enrollment expanding the neurosurgical workforce from 2010 to 2019. J Neurosurg. 2024;141(5):1427-1432. 38875717 10.3171/2024.4.JNS24158

[29] Cazorla-Morales IJ Chan AW Mikhail MM The Accreditation Council for Graduate Medical Education 20-year trends in diversity, equity, and inclusion in the United States: How does neurological surgery compare? World Neurosurg. 2024;185:e969-e975. 38458250 10.1016/j.wneu.2024.03.007

[30] Kabangu JL Lei C Yekzaman B Gender parity in neurosurgery residencies: an analysis. J Neurosurg. 2024;141(1):48-54. 38306646 10.3171/2023.11.JNS231152

[31] El Naamani K Reyes M Jreij G Women in neurosurgery: a cross-sectional demographic study of female neurosurgery residents in the United States. J Neurosurg. 2024;140(6):1785-1789. 38064710 10.3171/2023.9.JNS232080

[32] Pugazenthi S Malacon K Kim NC Barriers to neurosurgery for medical students: a national study focused on the intersectionality of gender and race. J Neurosurg. 2024;141(5):1395-1406. 38759239 10.3171/2024.2.JNS232038

[33] Wadhwa H Shah SS Shan J The neurosurgery applicant’s “arms race”: analysis of medical student publication in the Neurosurgery Residency Match. J Neurosurg. 2020;133(6):1913-1921. 31675693 10.3171/2019.8.JNS191256

[34] Holman L Stuart-Fox D Hauser CE. The gender gap in science: how long until women are equally represented? PLoS Biol. 2018;16(4):e2004956. 29672508 10.1371/journal.pbio.2004956 PMC5908072

[35] Thum JA Chang D Tata N Liau LM. Neurosurgeons in 2020: the impact of gender on neurosurgical training, family planning, and workplace culture. Neurosurg Focus. 2021;50(3):e11. 10.3171/2020.12.FOCUS20965 33789233

[36] Durham SR Donaldson K Grady MS Benzil DL. Analysis of the 1990-2007 neurosurgery residency match: does applicant gender affect neurosurgery match outcome? J Neurosurg. 2018;129(2):282-289. 29882698 10.3171/2017.11.JNS171831

[37] Gupta R Chen J Roth S Preresidency research output among US neurological surgery residents. J Neurosurg. 2024;141:63-71. 38427992 10.3171/2023.12.JNS231029

[38] Sheppard JP Lagman C Nguyen T Analysis of academic publishing output among 1634 successful applicants in the 2011-2018 neurosurgery residency match. J Neurol Sci. 2021;420:117186. 33223149 10.1016/j.jns.2020.117186

[39] Leschke JM Hunt MA. Electronic residency application service application characteristics associated with successful residency matching in neurosurgery in 2009-2016. World Neurosurg. 2018;113:e529-e534. 29477006 10.1016/j.wneu.2018.02.082

[40] Kistka HM Nayeri A Wang L Dow J Chandrasekhar R Chambless LB. Publication misrepresentation among neurosurgery residency applicants: an increasing problem. J Neurosurg. 2016;124(1):193-198. 26207605 10.3171/2014.12.JNS141990

[41] McGirt MJ Speroff T Dittus RS Harrell FE Asher AL. The National Neurosurgery Quality and Outcomes Database (N2QOD): general overview and pilot-year project description. Neurosurg Focus. 2013;34(1):e6. 10.3171/2012.10.FOCUS12297 23278267

[42] Asher AL Sammak SE Michalopoulos GD Time trend analysis of database and registry use in the neurosurgical literature: evidence for the advance of registry science. J Neurosurg. 2022;136(6):1804-1809. 34920432 10.3171/2021.9.JNS212153

[43] Conroy G. Surge in number of ‘extremely productive’ authors concerns scientists. Nature. 2024;625(7993):14-15. 38072985 10.1038/d41586-023-03865-y

